# Applying the trigger review method after a brief educational intervention: potential for teaching and improving safety in GP specialty training?

**DOI:** 10.1186/1472-6920-13-117

**Published:** 2013-08-30

**Authors:** John McKay, Carl de Wet, Moya Kelly, Paul Bowie

**Affiliations:** 1 Postgraduate GP Education, NHS Education for Scotland, Glasgow, UK; 2 General Practice & Primary Care, Institute of Health & Wellbeing, College of Medical, Veterinary and Life Science, University of Glasgow, Glasgow, UK

**Keywords:** Patient safety, General practice, Primary care, Trigger tool, Clinical record review, GP training, Clinical audit

## Abstract

**Background:**

The Trigger Review Method (TRM) is a structured approach to screening clinical records for undetected patient safety incidents (PSIs) and identifying learning and improvement opportunities. In Scotland, TRM participation can inform GP appraisal and has been included as a core component of the national primary care patient safety programme that was launched in March 2013. However, the clinical workforce needs up-skilled and the potential of TRM in GP training has yet to be tested. Current TRM training utilizes a workplace face-to-face session by a GP expert, which is not feasible. A less costly, more sustainable educational intervention is necessary to build capability at scale. We aimed to determine the feasibility and impact of TRM and a related training intervention in GP training.

**Methods:**

We recruited 25 west of Scotland GP trainees to attend a 2-hour TRM workshop. Trainees then applied TRM to 25 clinical records and returned findings within 4-weeks. A follow-up feedback workshop was held.

**Results:**

21/25 trainees (84%) completed the task. 520 records yielded 80 undetected PSIs (15.4%). 36/80 were judged potentially preventable (45%) with 35/80 classified as causing moderate to severe harm (44%). Trainees described a range of potential learning and improvement plans. Training was positively received and appeared to be successful given these findings. TRM was valued as a safety improvement tool by most participants.

**Conclusion:**

This small study provides further evidence of TRM utility and how to teach it pragmatically. TRM is of potential value in GP patient safety curriculum delivery and preparing trainees for future safety improvement expectations.

## Background

The Trigger Review Method (TRM) is a safety improvement approach which involves the rapid and structured review of clinical records by doctors and others clinicians [1-3]. The purpose is to screen these records for evidence of currently unknown patient safety incidents (PSIs) e.g. a patient presents with an adverse reaction to a medication which was not ‘allergy coded’ when a similar reaction had occurred previously. Where PSIs are detected learning needs, necessary corrective changes and opportunities for improvement are identified and actioned when judged appropriate.

The primary care version of the method involves sampling a small number of clinical records (c25) of a designated patient group or medical condition [1]. A clinical reviewer then screens each record searching for previously validated pre-defined ‘triggers’ (e.g. international normalized ratio >5.0 or abnormal blood result) which may be found in the record and which may point to the existence of an unknown PSI or latent risk to the patient (e.g. evidence that the patient was treated for an associated bleed or may have a developing malignancy).

The primary care TRM was adapted using previously published approaches in other health care sectors and developed and validated in Scottish general practices [1-4]. This TRM was developed using an educational paradigm to identify safety related learning needs and improvement opportunities and evolved from the traditional approach of quantifying acts of commission leading to harm. A small ‘proof of concept’ study established that the validated triggers were useful in helping GP reviewers to identify PSIs and latent risks [4]. Most detected incidents were of a low-to-moderate severity and judged to be preventable with the great majority of more serious incidents originating in secondary care settings but uncovered in primary care records. The TRM was subsequently tested as part of The Health Foundation-funded Safety & Improvement in Primary Care (SIPC) Pilot Programme in around 60 general practices in four territorial Health Boards in Scotland [5,6]. This programme was based on the collaborative model recently popularized by the Institute for Healthcare Improvement (IHI) but for the TRM involved additional one to one learning and practice-based support for participating teams. The programme evaluation suggested that the TRM has value as an important educational and improvement tool by enabling previously undetected threats to patients to be uncovered in the clinical record thereby providing the GP team with a new perspective on how to make patient care safer [5].

From a health care policy and implementation perspective, it was agreed during 2012 that Trigger Review evidence could be submitted as a Quality Improvement Activity as part of GP Appraisal in Scotland. A number of territorial Health Boards have financially incentivized general practices to voluntarily participate in Trigger Review through the provision of local enhanced financial contracts. The TRM is also a key part of the Scottish Government’s patient safety programme for primary care that was launched in March 2013 and is to be implemented nationally [7], while the Royal College of General Practitioners (RCGP) has included the method as one potential evidence source for revalidation purposes, but without any specific guidance on the approach or format [8].

In terms of regulatory and educational policy in the United Kingdom, ‘safety and quality’ is one of four professional domains describing the expected duties and standards of every doctor registered with the General Medical Council (GMC) [9]. Specifically, registered doctors are expected to ‘…*take part in systems of quality assurance and quality improvement to promote patient safety by: (a) participating in regular reviews and audit of the standards and performance of… work…taking steps to remedy any deficiencies [and] (b) regularly reflecting on the standards of practice and care you provide…*’ [9].

In response, medical educators have integrated patient safety-related topics and issues into undergraduate education [10] and specialist training programmes [11]. The UK Royal College of General Practitioners (RCGP) – which has responsibility for the content of the specialty training curriculum - has developed a curriculum statement on ‘patient safety’ [12]. Specific learning objectives are also defined which require the trainee to demonstrate a whole range of problem-solving skills aimed at improving the management of clinical risk and enhancing the patient experience of care (Table 1).

**Table 1:** RCGP curriculum learning outcomes (with examples) related to patient safety*

	**Learning outcome**	**Example**
1.	Primary Care Management	Contribute to the regular significant event audit (SEA) meetings and observe the benefits of a multidisciplinary team
2.	Person-Centred Care	Communicate openly, listen to and take patients’ concerns seriously and consider patient issues when reflecting on consultation experiences
3.	Specific Problem-Solving Skills	Demonstrate an awareness of the limitations of your own skills in risk management and illustrate that you understand when the skills of colleagues trained more extensively in risk management should be called upon
4.	A Comprehensive Approach	Describe the risks to patient safety by considering an illness pathway/journey in which a variety of healthcare professionals have been involved
5.	Community Orientation	Describe how patient groups may be put at increased risk of mishap by virtue of their particular characteristics, such as language, literacy, culture and health beliefs
6.	A Holistic Approach	Describe how the lessons of patient safety can be applied prospectively to doctor–patient interactions, especially through the identification and discussion of risk
7.	Contextual Aspects	Describe the impact of the working environment on the care the doctor provides and the likelihood of adverse incidents as a result of this
8.	Attitudinal Aspects	Help to shape an organisational culture that prioritises safety and quality through openness, honesty, shared learning and continual incremental improvement
9.	Scientific Aspects	Describe the basic principles of risk assessment

*UK GP Specialty Trainees are required to spend 18 months in a GP setting as part of a 3 or 4 year programme. The teaching required is governed by the RCGP curriculum and one area that is increasingly being highlighted is UK general practice is patient safety.

An assumption underlying much of the aforementioned initiatives is that all general practitioners and specialty trainees possess the requisite knowledge, skills and attitudes necessary to routinely apply safety improvement interventions, including TRM. And that in doing this they are able to apply the technique correctly, produce robust data, evaluate their findings and then plan and implement meaningful and sustainable improvements. A further assumption is that health and educational authorities are able to up-skill the GP workforce in TRM on the scale necessary to support the proposed implementation of this approach on a national basis.

However, a major disadvantage of the different stages of pilot development and testing of TRM in Scotland thus far is that GP participants (whether as part of the SIPC or independent practice based learning) received intensive 3-hour face-to-face training on their own clinical information systems by a visiting GP expert in this area. It is evident that this approach is costly and is neither feasible nor sustainable at the scale necessary. Attempts at developing a ‘train-the-trainer’ approach with other GP colleagues and educators have had minimal impact again because of the substantial time and financial costs this incurs. A key problem is that we have growing evidence that TRM is a valuable patient safety intervention in the areas of general practice in which testing has taken place, but we have difficulty in building the capacity and capability of the GP workforce to enable them to apply the technique successfully and with confidence.

Given the potential contribution of the TRM to professional and practice-based learning in making patient care safer, it is evident that the approach may have a role to play in specialty training to help prepare GP trainees for the contractual and regulatory demands of independent clinical practice. This is particularly so given the proposal to extend specialty training to a four-year programme which will include the need to undertake a quality improvement project with TRM being a potential tool for this purpose [13]. The training environment would also allow us to test the impact of a low cost and less intensive educational intervention using conventional teaching methods which could potentially be delivered at scale and by non-clinical educators. Against this background, we aimed to determine the acceptability, feasibility and potential impact of TRM and a related educational intervention in the GP training environment.

## Methods

### Study design

We trained a small number of volunteer GP specialty trainees to apply the TRM and asked each one to undertake a structured review of specific samples of clinical records in their training practice and to document their findings. The clinical records were defined as the electronic patient record sections relating to: clinical encounter entries, repeat and acutely prescribed medication, correspondence with secondary care and other relevant organizations, clinical investigations (such as blood test results) and medical record READ codes for diseases and allergies. The review period was defined as the last three consecutive calendar months in each record. This period of time arguably offers the most efficient compromise between yield of triggers and time spent by the healthcare professional examining the notes [1].

### Sample and setting

In April 2012, we emailed all GP Educational Supervisors in the west of Scotland Deanery with details of the proposed pilot study and asked them to discuss with their trainees the possibility of volunteering to participate. The numbers of participants were restricted to the first 25 GP trainees to indicate a willingness to participate via email response.

### Training intervention

We held a two-hour workshop training session on two occasions in May 2012 in a central Glasgow location to give some attendance flexibility to the volunteer participants. The two training sessions were delivered by the same facilitators (PB and JM) and consisted of a range of basic educational methods and components which are summarized in Table 2. The main aims were to clarify study expectations, raise awareness of patient safety issues in general practice, introduce the concept and role of TRM, and help prepare participants to apply the method in their own practices and report back findings. Participants were given the opportunity to independently conduct a trigger review of a pre-prepared simulated full patient record (which contained ‘planted’ evidence of PSI scenarios) and then discuss this in a group work session. The set objective was to identify at least four of the PSIs contained therein within 20 minutes and to make judgements on the severity and preventability of these incidents, if detected.

**Table 2:** Components of the basic training intervention

**n**	**Component**
1.	A short Powerpoint *presentation* about the Trigger Review Method (TRM) and its role in the Safety Improvement in Primary Care (SIPC) Programme;
2.	A *patient safety quiz* with feedback about the evidence base for error and safety in general practice;
3.	A *group work exercise* on matching a range of risk and safety terms to the provided definitions to help increase participants’ shared understanding of key terms, including ‘patient safety incident’;
4.	Hand-out and demonstration of a TRM *educational support package* consisting of: (i) step-by-step guidance; (ii) simulated patient records with ‘worked out’ solutions; (iii) the Trigger Review Summary Report (TRSR); and (iv) a description with practical examples of how patient safety incidents’ severity and preventability should be rated;
5.	A *practical exercise for individual participants* to perform a ‘trigger review’ of a simulated patient record followed by discussions in small groups and then in an open forum;
6.	Clarification of the study’s expectations of the GPSTs by informing them of the ‘high risk’ patient groups from which they were to select their sample of clinical records for trigger reviews and where to send their completed TRSR documentation.

### Selection of clinical records to review

To further test the applicability of the TRM, we asked the first group of participants (attendees of the first learning session) to sample records from a single patient group (patients aged over 75 years with Ischaemic Heart Disease - IHD). Those attending the second learning session were asked to select any patient group that they judged to be ‘high risk’ or relevant, whether on the pre-defined list (Additional file 1) or otherwise.

### Data collection

We asked each trainee to select and review a recent three-month period in each of 25 clinical records from their study populations and to complete a Trigger Review Summary Report (TRSR) electronically or in writing. The TRSR is a 2-page pro-forma to collect and summarize data on the number of detected ‘triggers’, details of any PSIs uncovered, learning needs identified and actions that were or should be taken as a result of the review process.

Participants were strongly encouraged to ‘fix’ any obvious problems that could be achieved quickly and without much effort e.g. updating a patient’s allergy coding status. However, for the sections on the TRSR concerned with reflection, learning and improvement, trainees were asked to simply outline how they would (hypothetically) act on their findings rather than actually undertake any of these recommendations because of the additional time and commitment this may entail.

Participants were advised that the total review process, including completion of the TRSR should take approximately 2–4 hours and that no single record should take more than 20 minutes to review. Trainees were given a four-week period after their training session to undertake the TRM and submit completed TRSR documentation to the study leaders.

### The trigger review summary report (TRSR)

The TRSR was designed by the authors and is a product of four years of research, and practical experience of training and applying the TRM in general practice settings in Scotland (Additional file 2). The report content is aligned closely with the reflection, learning and improvement expectations of GP appraisal and specialty training.

The National Patient Safety Agency’s definition of a PSI is included on the TRSR to help guide reviewers when screening the clinical record: “*any unintended or unexpected incident which could have or did lead to harm for one or more patients receiving NHS care*“ [14]. This is useful because from a clinical risk and patient-centered perspective the key focus is on detecting a circumstance where harm occurred (physically or psychologically and regardless of severity) or could have happened but was prevented (a near miss) or could happen at some point in the future (a latent risk). The focus of the TRM and TRSR are therefore preventable patient safety incidents.

We designed a dual scoring system to allow reviewers to judge the perceived severity and preventability of detected PSIs and included this on the TRSR. The severity classification system was adapted from previously published work to suit the general practice context [1-4]. As far as we know, there is a lack of published guidance on how to judge the ‘preventability’ of detected patient safety incidents. We therefore co-developed and agreed a basic preventability rating scale to help participants make this professional judgement. Combining the severity and preventability ratings (scales from 1 to 4) provide a single ‘priority score’ (scale from 2 to 8). This score may aid clinicians in prioritizing incidents for learning and improvement in the event that an unmanageable number of incidents are detected i.e. incidents with higher scores should be prioritized over incidents with lower scores.

### Experiences and perceptions of TRM

After completing their record reviews and submitting TRSR documentation, participants were invited to attend a two-hour facilitated workshop in July 2012 to reflect on the process. The workshop explored trainees’ experiences and perceptions of the acceptability, feasibility and potential usefulness of the trigger review approach. Discussions were recorded on a mobile recording device with the consent of participants and contemporaneous notes were also taken.

### Data analysis

The data from the submitted TRSR were anonymized, extracted and coded in an Excel spreadsheet and included: (i) the numbers of individual triggers identified; (ii) the severity and preventability ratings of Patient Safety Incidents; (iii) learning points identified from the TTR and; (iv) actual or proposed actions resulting from the review. Quantitative data were analyzed using simple descriptive statistical methods in Microsoft Excel. The qualitative data from the TRSR were transcribed and grouped by section. The authors independently re-played the workshop recording on an iterative basis and compared their notes and TRSR data before discussing and agreeing the main related themes which best reflected the discussion. Key insights on the perceived acceptability, feasibility and impact (or otherwise) of the TRM are presented.

## Results

### Response rate

21/25 trainees (84%) who attended the training sessions undertook trigger reviews of their clinical records and submitted completed TRSR documentation within the requested 4-week time period.

### TRM findings

A total of 520 unique patient records were reviewed by all participants who identified 468 triggers. The frequencies, means and ranges of all the identified triggers are shown in Table 3. The most commonly detected trigger was ‘frequency of consultations’ (n = 119, mean 5.67, range 0–20) and the least common was the ‘optional triggers’ (n = 9, mean 0.42, range 0–14).

**Table 3:** **The number of detected triggers, patient safety incidents and intended actions reported by GP trainees in groups 1** & **2 and overall**

	**Group 1 (n = 14)**		**Group 2 (n = 7)**		**All (n = 21)**	
	**No**	**Mean (range)**	**No**	**Mean (range)**	**No**	**Mean (range)**
**Triggers**						
≥3 Consultations	83	5.9 (0–14)	36	5.14 (0–20)	119	5.67 (0–20)
New ‘high priority’ code added	45	3.2 (0–12)	29	4.14 (0–9)	74	3.52 (0–17)
New allergy code	15	1.0 (0–3)	7	1.0 (0–4)	27	1.05 (0–4)
Repeat medication item discontinued	46	3.3 (0–9)	17	2.43 (1–6)	63	3.00 (0–9)
Out-of-Hours/A&E attendance	51	3.64 (0–8)	19	2.71 (1–5)	70	3.33 (0–8)
Hospital admission	46	3.29 (0–6)	19	2.71 (0–6)	65	3.10 (0–6)
Hb < 10.0	16	1.14 (0–6)	1	0.14 (0–1)	17	0.81 (0–6)
eGFR reduction ≥5	22	1.57 (0–11)	2	0.29 (0–1)	24	1.14 (0–11)
Optional triggers	8	23 (0–14)	1	0.14 (0–1)	9	0.43 (0–14)
	**N = 14**		**N = 6**		**N = 20**	
**Patient safety incidents**	62	4.4 (2–9)	18	4.0 (1–5)	80	4 (1–9)
*Severity*						
1	13	0.93 (0–3)	4	0.67 (0–2)	17	0.85 (0–3)
2	21	1.5 (0–3)	7	1.12 (0–2)	28	1.4 (0–3)
3	15	1.07 (0–2)	3	0.5 (0–1)	18	0.9 (0–2)
4	13	0.93 (0–5)	4	0.67 (0–2)	17	0.85 (0–5)
*Preventability*						
1	4	0.29 (0–2)	1	0.17 (0–1)	5	0.25 (0–2)
2	30	2.14 (0–4)	9	1.5 (0–4)	39	1.95 (0–4)
3	17	1.21 (0–5)	5	0.2 (0–2)	22	1.1 (0–5)
4	11	0.78 (0–3)	3	0.6 (0–1)	14	1.65 (0–3)
*Priority*						
2	1	0.07 (0–1)	0	0.33 (0–2)	1	0.05 (0.1)
3	8	0.57 (0–2)	3	0.5 (0–2)	11	0.45 (0–2)
4	13	0.93 (0–2)	1	0.17 (0–1)	14	0.7 (0–2)
5	15	1.1 (0–2)	9	1.5 (0–3)	24	1.2 (0–3)
6	14	1.0 (0–3)	4	0.8 (0–1)	18	0.9 (0–3)
7	8	0.07 (0–3)	1	0.17 (0–1)	9	0.45 (0–3)
8	3	0.21 (0–5)	0	0	3	0.15 (0–5)
**Intended next actions**						
Significant event analysis	8	0.57 (0–3)	2	0.33 (0–2)	10	0.5 (0–3)
Clinical audit	9	0.64 (0–2)	1	0.17 (0–1)	10	0.5 (0–2)
PDSA cycle	2	0.14	0	0	3	0.15 (0–2)
Feedback to colleagues	20	1.43 (0–5)	9	1.5 (0–4)	29	1.45 (0–5)
Make a specific improvement	8	0.57 (0–2)	0	0	8	0.4 (0–2)
Add to appraisal documentation	19	1.36 (0–5)	0	0	19	0.95
Discuss with Educational Supervisor	26	1.86 (0–8)	2	0.33 (0–1)	28	1.4 (0–8)
Protocol update	6	0.43 (0–3)	1	0.17 (0–1)	7	0.35 (0–3)

Participants reported a total of 80 previously undetected PSIs (15.4%). The 14 trainees in Group 1 (patients with IHD and >75 years old) reviewed the clinical records of 345 patients (mean records 24.6; range: 15–30), detecting a total of 62 PSIs (18%; mean 4.4; range: 2–9). The 7 trainees in Group 2 (IHD, COPD and housebound patient populations) reviewed the clinical records of 175 patients, detecting a total of 18 PSIs (10.3%; mean 4.0; range: 1–5) while one respondent did not find any. Examples of detected PSIs are shown in Table 4.

**Table 4:** Examples of detected patient safety incidents* judged to be preventable or potentially preventable

•	Failure to initiate prophylactic treatment for or follow up a patient with gout resulted in a hospital admission
•	Patient with a significant drug allergy [prescribed the same medication] resulting in a further allergic reaction
•	‘Patient given inappropriate dosages of anti-diabetic medication with resultant renal injury’
•	‘Patient’s [misunderstanding] of warfarin dose led to increased requirement for monitoring’
•	‘Delayed diagnosis of ischaemic heart disease led to avoidable admission’
•	‘Lack of monitoring LFTs of a patient taking anti-fungal medication [resulted in] intensive follow-up’
•	Patient became symptomatically bradycardic as a result of a drug known to have this side effect and required review and medication adjustment
•	‘Change in medication with known side effects [may have] resulted in a fall and hospital admission’
•	Hospital admission for abdominal problem (overflow) due to incomplete assessment of patient by primary healthcare team
•	A delay in monitoring after an increased dosage of nephrotoxic medicine leading to a significant decrease in renal function – with increased monitoring requirements
•	Admission for transfusion from potentially avoidable delay in monitoring
•	Potential delayed diagnosis in symptomatic atrial fibrillation leading to hospital admission

*Some incidents occurred more than once or were detected by more than one respondent. The original phrasing of some patient safety incidents were reworded to aid clarity.

The perceived rating of the severity, preventability and ‘priority’ accorded to these detected PSIs are shown in Table 3. 36/80 of incidents (45%) were judged to be preventable or potentially preventable and 35/80 were judged to have resulted in moderate to severe harm (44%). Incidents were given priority numbers from 2 to 8, with ‘5’ the most common (n = 24, mean 1.2, range 0–3) and ‘2’ the least with a single incident.

### Learning needs and learning points

The majority of participants were able to identify learning needs at the personal, professional and wider practice team level (Table 5). Many individual learning needs concerned chronic disease management. At the practice level trainees reported perceived needs for improved communication between primary and secondary care, consistent coding and ‘protocols’ for specific high risk responsibilities (e.g. monitoring of nephrotoxic drugs). Some participants reported that they had recognized important learning points and their responses indicated reflection on their actions. For example, one trainee reported the intention to *‘…action more thorough medication reviews…’* while another wrote about recognizing ‘…*the importance of coding as a safety issue*…’ A further selection of learning needs and points are provided verbatim in Table 5.

**Table 5:** A selection of personal, professional and practice learning needs and points identified and reported by GPSTs

**Personal and professional learning needs**	
•	‘Review SIGN and NICE cardiovascular heart disease guidelines’ and ‘Need to update [my] knowledge on management and therapeutics of heart failure’
•	‘Need for new knowledge on gout management’
•	‘[Find out] how to liaise with social services about respite [care]’
•	‘How different Quality improvement (QI) techniques can be used’
•	‘Need to examine previous clinical notes to identify root of potential difficulties [that caused the detected patient safety incidents]’
•	‘[What are the patient] self management issues in COPD
•	‘Revise indication for warfarin in atrial fibrillation’
**Learning needs for the practice team**	
•	‘Need to update diabetic guidelines on therapeutics and management’
•	‘Need system for dealing with out of hours (OOH) mail’
•	‘Need system for better medication reviews and monitoring’
•	‘Need for [consistent] adverse event coding’
•	‘Need to develop protocol for falls prevention’
•	‘Need to develop more continuity in patient care’
•	‘Address appointment availability’
•	‘Examine how hospital discharge prescriptions are actioned’
•	‘How to highlight medication errors to allow action’
•	‘To improve communication within primary care team’
•	‘How to carry out quality improvement techniques’
•	‘How to do trigger review’
•	‘Protocol for monitoring potential nephrotoxic [and hepatotixic] drugs’
**Learning points**	
•	‘[I realized the] importance of coding as a safety issue’
•	‘[I] need to give more attention to out of hours summary sheets’
•	‘Need to action more thorough [medication] reviews’
•	‘How to carry out searches [to identify specific patient populations in the practice]’
•	‘[I need to] revise medication interactions’
•	‘[What are the] potential high yield triggers to identify problems’
•	‘[What] factors are involved (medical & social) in warfarin prescribing’
•	‘Recognition of the ‘cascade of error’ and need for root cause analysis’
•	‘Positive learning that disease monitoring systems work well (COPD) [in this practice]’

### Improvements and intended next steps

Participants reported performing many, diverse actions during the review with the most common involving medication e.g. reviewing prescribed items, amending dosages or arranging for monitoring through further blood tests. A selection of practical changes implemented during the review process is shown in Table 6.

**Table 6:** A selection of actions and improvements undertaken by trainees during the review process

•	‘Potential nephrotoxic and cardiotoxic medication discontinued’
•	‘Drug dosage (warfarin) adjusted’
•	‘Referral letter to secondary care done’
•	‘Allergy, adverse drug reaction and clinical procedure codes entered or updated’
•	‘Case discussion with educational supervisor’
•	‘Medication reviews done / medication adjustments made’
•	‘Arranged a review appointment for a patient’
•	‘Updated notes with investigation results’
•	‘[Necessary] follow-up blood test arranged’
•	‘When I came to use it, I had to skill myself up in EMIS which was a good thing’
•	‘A pre-audit Tool to inform SEA and Audit topics [as] clinicians often stumped for topics’
•	‘Should use this approach with ST1s’
•	‘Good link with appraisal and revalidation’

The vast majority also indicated their intention to take further, specific actions after the review process (Table 1). The most common actions were to give feedback about the review findings to colleagues (n = 29, mean 1.45, range 0–5) and to discuss it with their educational supervisors (n = 28, mean 1.4, range 0–8), providing the basis for enhanced team-working on safety-related issues. They were least likely to consider applying the Plan-Do-Study-Act (PDSA) method (n = 3, mean 0.15, range 0–2) or to update a practice protocol (n = 7, mean 0.35, range 0–3).

### Perceptions of training intervention and TRM feasibility and impact

A selection of comments about the training intervention and the acceptability, feasibility and potential usefulness of the Trigger Review Method is shown in Table 7.

**Table 7:** A selection of comments and perceptions about the training intervention and the acceptability, feasibility and potential usefulness of the Trigger Review Method

**The training intervention**	
•	‘Positive experience’
•	‘Not aware of [the trigger review method] previously’
•	‘Case-based scenarios helped us to focus on what to look for, good idea to have a practice beforehand’
•	‘Good that we did it individually but could then ask questions of each other in our small groups’
•	‘Left confident that we could apply the process’
•	‘Matching the case record example to EMIS/Vision would be a big help’
•	‘Liked the handouts, good reference a few weeks later’
**Acceptability**	
•	‘Initially a bit annoying but good when you get into it’
•	‘More interesting when audit is your own and relevant to you’
•	‘Very good experience, sharing with colleagues and leading to further audit’
•	‘Too reticent to discuss uncovered issues with colleagues for fear of offending or upsetting, particularly given junior position’
•	‘[The TRM is a] good way of identifying important safety concerns’
**Feasibility**	
•	‘Focus needs to be on high risk groups’
•	‘Very simple and quick to go through - triggers can be done in 2 minutes’
•	‘Difficult for non-clinical staff, practice nurses might be even better, though might need GP guidance’
•	‘Duration of time taken ok’
•	‘Couldn't open electronic version’
•	‘Increasing sample size not a real issue as it's quick and easy to find triggers and review records where nil of note found’
**Potential usefulness**	
•	‘Highlighted many interface issues [e.g. secondary care], not following-up [or] informing us to follow-up [patients]’
•	‘Good to see all the potential, all the things we're doing to stop potential harm’
•	‘Helped to change our [practice] protocol’
•	‘Arguably more useful than audit, greater sense of ownership’

The majority of participants experienced the training session as a ‘…*positive learning experience….*’ They appeared to value the educational support material provided and the opportunity to practice the skill on a simulated record and to discuss the findings with the support of their small groups. While the majority were previously unaware of TRM, they generally ‘…*left confident that we could apply the process*…’

The TRM appeared acceptable to the vast majority of trainees. They appreciated that this quality and safety improvement method is applied in your own practice and that the findings are ‘…*your own and relevant to you*…’ and can inform further actions. However, one participant was concerned about ‘…*uncovering issues*…’ which may ‘…*offend or upset*…’ colleagues.

Trainees generally found the process ‘…*very simple and quick to go through*…’ The mean time spent reviewing records for Group 1 was 172 minutes (range: 25 to 500 minutes). The mean time spent reviewing records for Group 2 was 122 minutes (range: 45 to 180 minutes). Their subjective experience of this was described during the workshop as ‘…the duration of time taken was ok…’. The whole issue of ‘time taken’ did not appear to be problematic for this group of participants. One GPST struggled to open the electronic copy of the TRSR but was able to submit a written copy instead.

The vast majority of participants thought that TRM is potentially useful. They commented on the value of identifying latent system risks, but were also pleased to identify ‘…*all the things we’re doing to stop potential harm*…’ They considered the TRM as having at least as much value as other quality improvement methods and having the potential to complement existing tools such as significant event analysis (SEA) and clinical audit. In addition, they also thought the TRM would have a ‘…*good link with appraisal and revalidation*…’

## Discussion

To our knowledge, this was the first attempt to pilot TRM in the GP specialty training environment and to assess its potential as an educational and improvement intervention. Our small study found that the vast majority of trainees who underwent the streamlined training intervention were able to implement TRM, and detect small numbers of preventable PSIs, particularly in the high risk IHD elderly patient group. All participants were able to demonstrate some element of reflection, document potential learning needs and develop improvement action plans regardless of population group audited.

The findings reaffirm the potential of this method to identify largely minor but avoidable PSIs which nonetheless have educational and improvement value, particularly in *high risk* groups of patients. In addition, most participants experienced the method as acceptable and feasible and perceived it as potentially useful. However, some appeared to misapply aspects of the method, while others selected patient groups (e.g. housebound patients) which by definition are likely to yield limited information on clinical risks and the potential for capturing patient safety-related issues - this has allowed us to further update how future practitioners are trained in applying the technique. Importantly, our ‘new’ group training intervention appeared to work in the sense that the majority of participants were able to implement TRM and report findings that are consistent with previous studies which used the more intensive – but less feasible - one-to-one teaching approach.

The most and least frequent reported ‘interventions’ were non-specific discussions with a colleague or trainer and conducting a plan-do-study-act (PDSA) cycle (PDSA is included in the TRM form because a minority of GPs have previous experience of this approach as part of national QI programmes). For trainees in this study, it is highly likely that there is a lack of knowledge of this specific QI method since there is no formal training on this approach within the current GP curriculum. But this would not explain the relatively low numbers indicating their intention to undertake the other QI methods mentioned (audit and SEA) which are formally taught. From a human error and systems perspective of patient safety this is likely to indicate a major learning need - for trainers and trainees - to gain much greater knowledge of those complex human-system interaction issues which frequently contribute to PSIs. This understanding is necessary before any appropriate improvement intervention can be decided upon to improve system reliability and so mitigate risks and reduce the potential for patient harm.

### Comparison with existing literature

Similar to evidence of significant event analysis (SEA) and clinical audit implementation in general practice, this small study suggests some variations in how the technique is understood and applied [15-17]. Consequently, this impacts on how useful it may be in identifying latent risks and safety issues of professional and organisational interest and, most importantly, the learning and improvement value to be gained from applying the method.

Perhaps one of the strongest issues arising from trigger review is the identification of incidents that can serve as topics for SEA and Audit e.g. delayed diagnoses, sub-optimal therapeutic management, poor disease and drug monitoring, and the appropriate use of Information Technology. This is particularly helpful given that appraisal and revalidation requires GPs to analyse two significant events per year (with the GMC encouraging these events to be PSIs rather than broader quality of care issues). Identification and analysis of these previously undetected PSIs is particularly pertinent to improving the opportunity cost of SEA topics.

A factor associated with TRM is that the most severe events detected tend to be secondary care generated [4], which are the most difficult for primary care clinicians to address or influence. Our study shows that the identification of moderate severity PSIs by trainees allows them to tackle issues that can be addressed by themselves and the practice team, which in turn allows them to demonstrate leadership qualities and teaching skills now expected of the training curriculum learning outcomes [12,18].

### Practical implications of findings

It is likely that in the training environment simply considering the validated triggers when reviewing clinical records is a useful starting point for developing and teaching the technique. The group teaching format was judged acceptable by participants hence improving the potential feasibility of delivering teaching to larger numbers of trainees (and other clinicians) in comparison to one-to-one teaching (by another clinician) for specific aspects of the training curriculum. This is in keeping with other QI techniques such as criterion audit and SEA which can be taught by both clinicians and non-clinicians in large group settings but applied at the individual and practice-based levels. The issue of prolonged time taken to apply the method by a minority of respondents and the lack of findings of patient safety incidents for some, may point to areas for improvement in terms of teaching by the development team or application by the trainee.[19] Alternatively, it is possible that no PSIs were actually present and that time was a issue because of the complexities of the cases being studied.

The technique allows the identification of PSIs arising in general practice and the findings suggest that these are directly related to issues within the practice which enables focused discussion with educational supervisors and other colleagues, potentially facilitating rapid implementation of learning and remedial actions. It may also serve as a substrate for both formative and workplace case-based discussions and assessments.

### Strengths and limitations

Our sampling strategy and small sample size were pragmatically informed by available time, resources and ready access to an established network of general practices with training status and their trainees. For feasibility issues (principally time as the GPST year was effectively completed within two weeks of the final submission dates in our study) we were unable to follow-up the five trainees who attended training workshops to ascertain the reasons why they were unable to undertake the trigger review and submit completed documentation to us. Knowing this would have provided us with greater insights into the overall utility of this method and associated implementation issues, particularly the time taken for review and the subsequent yield of PSI. Volunteers may not, therefore, be representative of trainees in general. All data were reported to us so we have no means of independent verification. Other clinical reviewers may have arrived at different judgements in terms of whether a situation was indeed a PSI, how severe it was and whether it was preventable – the reported data may therefore be an over or an under representation. All findings should be viewed within these contexts.

As far as we are aware, there is a lack of published guidance on how to judge the ‘preventability’ of detected PSIs. This is a critical and often overlooked issue in the patient safety literature: unfortunately but inevitably patients will be unavoidably harmed as a result of their interactions with healthcare for a range of highly complex reasons. The key focus from the patient’s and the clinician’s perspective should be on detecting and learning from those incidents which are judged to be preventable i.e. there is consensus that they should not have occurred if the appropriate preventative strategies had been in place. We therefore co-developed and agreed a basic preventability rating scale to assist participants in making this professional judgement.

The ‘priority score’ (scale from 2 to 8) was intended to aid clinicians in prioritizing incidents for learning and improvement in the event that an unmanageable number of incidents are detected, e.g. incidents with higher scores should be prioritized over incidents with lower scores. Pragmatically, it is possible that GP teams would not be able to feasibly deal with all incidents detected during a review, given time and resource constraints, so this process is designed to help clinicians prioritise future actions if they choose to activate this option. However, although participants appeared to understand why this may be important, we did not find evidence of its practical usefulness in this pilot study, perhaps because of the low numbers of preventable PSIs detected and the perceived need to prioritize.

A further systems issue is whether GPs are prepared to highlight and discuss PSI’s identified through TRM with secondary care. It is known that some GP’s will deal with such interface issues if they identify a significant event [16] but this is neither compulsory or adequately formalized.

### Further implementation

At deanery level next steps will be to further refine TRM as a tool together with the associated training process and educational supporting materials. Exploring the potential need for e-learning and other interactive technology will also be necessary. The short term aim is to spread and evaluate this approach within the regional training environment (by encouraging GP educational supervisors to use the brief intervention developed) before bringing it to the attention of other Scottish medical deaneries with a view to national implementation. However, there is a pressing need to raise awareness of TRM amongst GP educational supervisors and training programme directors to demonstrate the links with the forthcoming national patient safety programme, GP appraisal and future contractual incentives. Participation in TRM prepares the trainee for the ‘real world’ from the perspective that it can be used as quality improvement for appraisal and may be the basis of the proposed QI project as part of enhanced training as envisaged by RCGP [12]. Simultaneously it meets the expectations for the RCGP curriculum for patient safety in terms of the focus on managing risk, taking a patient-centered approach to care, and demonstrating team working and leadership [12].

For wider general practice, a key learning point of this study is the potential to quickly build capacity and capability using the basic teaching methods outlined. All Scottish territorial boards will be expected to implement the national patient safety programme and support their primary care workforces educationally in doing so. The TRM educational materials and ‘guide sheets’ developed over the past few years and further refined in this study will be made freely available to local clinical (and non-clinical) educators and quality improvement facilitators to enable them to adapt and use these to suit contexts.

## Conclusions

This small study provides further evidence for the utility of the trigger review method as a safety improvement intervention and also describes one way to teach this approach pragmatically in primary care settings. The findings demonstrate the potential value of TRM in the GP specialty training environment in helping to deliver the patient safety curriculum and also in preparing GPs to meet current and future educational, contractual and regulatory quality improvement expectations.

### Ethical review

The study did not require ethical approval – judged to be service evaluation.

## Competing interests

The authors declare that they have no competing interests.

## Authors’ contributions

CdW and PB conceived the study idea. JM and PB delivered the training interventions and facilitated the workshop feedback session. CdW developed the supporting educational resources. JM collected and analyzed the data. CdW and PB co-drafted the initial manuscript. JM and MK further reviewed the manuscript and added additional material and clarifications. All authors contributed equally to the preparation of the final paper. All authors read and approved the final manuscript.

## Pre-publication history

The pre-publication history for this paper can be accessed here:

http://www.biomedcentral.com/1472-6920/13/117/prepub

## Supplementary Material

Additional file 1 Trigger Review of Electronic Patient Records – Examples of potential patient sub-populations to audit. Click here for file

Additional file 2 The Trigger Review Summary Report Template. Click here for file
