# Supplementary material for: Applying the trigger review method after a brief educational intervention: potential for teaching and improving safety in GP specialty training?
Source: BMC Med Educ. 2013 Aug 30;13:117. doi: 10.1186/1472-6920-13-117 (PMC3846442; doi:10.1186/1472-6920-13-117)
Supplement: Additional file 1 — Trigger Review of Electronic Patient Records – Examples of potential patient sub-populations to audit. [file 1472-6920-13-117-S1.doc]

NHS Education for Scotland

**Trigger Review of Electronic Patient Records**

**EXAMPLES OF POTENTIAL PATIENT SUB-POPULATIONS TO AUDIT***

| **1. Specific, Shared Patient Characteristics** | **2. Chronic Disease** | **3. High Risk Medications** |
| --- | --- | --- |
| Nursing Home Patients | COPD | Insulin |
| >75 years | Stroke/TIA | Morphine |
| Last 25 Attending Out-of-Hours | CVD | Warfarin |
| Last 25 Hospital Referrals | Diabetes | NSAIDs |
| Housebound patients | Heart failure | Diuretics (x2) |
| Last 25 Hospital Admissions | CKD | >5 Repeat medication items |
| 1. **Combinations of Groups 1 to 3**   e.g. Patients over 75 years, taking 5+ medications, who attended in previous 12-weeks; nursing home patients prescribed NSAIDs; patients with heart failure and who are prescribed 2 or more diuretics. | | |
| 1. **Choose Your Own Sub-Populations**   e.g. Patients discharged after emergency hospital admission (review the period before and after admission), a random selection of any 25 patients registered with the practice. | | |

*Patients’ susceptibility to patient safety incidents varies widely and is influenced by many factors including age, frequency of consultation, co-morbidities and the number and types of prescribed medications. The rationale for choosing a specific sub-population of patient records to review is that it increases the likelihood of detecting patient safety incidents. There is no single ‘right’ group to choose. In practice, the selected patient groups will mainly depend on the reviewers’ preference and review aims.
