# Supplementary material for: Applying the trigger review method after a brief educational intervention: potential for teaching and improving safety in GP specialty training?
Source: BMC Med Educ. 2013 Aug 30;13:117. doi: 10.1186/1472-6920-13-117 (PMC3846442; doi:10.1186/1472-6920-13-117)
Supplement: Additional file 2 — The Trigger Review Summary Report Template. [file 1472-6920-13-117-S2.pdf]

Trigger Review Summary Report

NHS Education for Scotland

9864344862

Step One: Planning and Preparation

**Name of Reviewer:**	
**Name of Practice:**	
**Profession:**	☐ GP Partner ☐ GPST ☐ Locum/Sessional GP ☐ Salaried GP ☐ Practice Nurse
**Date of Review:** *(in dd/mm/yy format)*	/ /
**Review Period:** *(e.g. 3 calendar months)*	Please select
**What Patient Group did you select records from?**	

Step Two: Review of Records

Please aim to review **25** records from the chosen patient group. Tick one box (✓) next to each trigger, each time you find it in one of the records. The number of boxes is NOT related to the number of records.

**Trigger** *(a 'prompt' that may indicate a safety incident)*		Total
≥ 3 consultations in 7 days		0
New 'high' priority read code added		0
New allergy read code added		0
'Repeat' medication item discontinued		0
Out of Hours / A&E attendance		0
Hospital admission		0
Hb <10.0		0
eGFR reduction ≥ 5		0
Optional Trigger		0

Review of Findings

Please briefly describe the patient incidents that you detected. Next, judge the severity and preventability of each incident using the scales below.

	Description of Detected Patient Safety Incidents*	Severity		Preventability		PRIORITY
1			+		=	0
2			+		=	0
3			+		=	0
4			+		=	0
5			+		=	0

***Patient Safety Incident:** *"Any incident that caused harm, or could have caused harm to a patient as a result of their interaction with health care"*
(The definition encompasses error, harm, adverse event, significant event and near miss)

Severity Scale	Preventability Scale
1 Any incident with the potential to cause harm.<br>*2 Mild harm: inconvenience, further follow-up or investigation to ensure no harm occurred.*<br>*3 Moderate harm: required intervention or duration for longer than a day.*<br>*4 Prolonged, substantial or permanent harm, including hospitalisation.*	*1 Not preventable and originated in secondary care.*<br>*2 Preventable and originated in secondary care OR not preventable and orginated in primary care.*<br>*3 Potentially preventable and originated in primary care..*<br>*4 Preventable and originated in primary care.*

© 2012 NHS Education for Scotland

Step Three: Reflection, Action & Improvement

A. Please describe any Actions/Improvements made DURING the review (e.g. updated coding, reviewed prescribing)

B. What do you plan to do NEXT as a result of the trigger review findings? Use the 'priority' scores as a guide if relevant. Tick as many action boxes below as appropriate for each detected inicident. Write a brief description of the planned actions or add any actions not covered by the suggestions below.

Specific Actions	1	2	3	4	5	Please describe:
Significant event analysis	☐	☐	☐	☐	☐	
Audit	☐	☐	☐	☐	☐	
PDSA Cycle	☐	☐	☐	☐	☐	
Feed back to colleagues/GP Trainer	☐	☐	☐	☐	☐	
Make a specific improvement(s)	☐	☐	☐	☐	☐	
Add to Appraisal documentation	☐	☐	☐	☐	☐	
Submit a formal incident report	☐	☐	☐	☐	☐	
Update or develop a protocol	☐	☐	☐	☐	☐	
Other:	☐	☐	☐	☐	☐	

C. Please describe identified Personal, Professional or Practice Team Learning Needs:

Personal:

Professional:

Practice Team:

Please add any comments about the trigger review process

Approximately what length of time (in hours) did the review and completing this report take? Please select

© 2012 NHS Education for Scotland
